# COVID-19 Presenting as Acute Icteric Hepatitis

**DOI:** 10.7759/cureus.16359

**Published:** 2021-07-13

**Authors:** Warren R Balaja, Sarah Jacob, Soheila Hamidpour, Amgad Masoud

**Affiliations:** 1 Internal Medicine, University of Missouri Kansas City School of Medicine, Kansas City, USA; 2 Pathology, University of Missouri Kansas City School of Medicine, Kansas City, USA

**Keywords:** covid-19, jaundice, icterus, acute hepatitis, case report

## Abstract

As the coronavirus pandemic continues to evolve, so does the understanding of different presentations of disease. In this case report, we describe a patient whose presentation of COVID-19 was with acute icteric hepatitis without respiratory symptoms. This is the first case in the literature to our knowledge to report jaundice as the initial presentation of disease and adds to just a handful of cases in the literature of acute hepatitis as the sole presentation of COVID-19. Additionally, despite severe hepatitis, the patient had a benign course of COVID-19 and did not require aggressive medical care; this strays from conventional paradigms that associate severity of COVID-19 with a degree of aminotransferase elevation. The purpose of this report is to make physicians aware of acute icteric hepatitis as a presentation of COVID-19 infection and to facilitate discussion and further research in the area of COVID-19-induced hepatitis.

## Introduction

The COVID-19 pandemic has caused more than 139,000,000 global cases and more than 2,900,000 deaths as of April 2021 since its origination in December 2019 [1]. The clinical course can vary with age and comorbidities but typically presents with respiratory symptoms such as fever, cough and malaise. Other less common symptoms include sore throat, chest pain, chills, nausea, and vomiting [2]. Most people see signs and symptoms five days after infection but some can present up to two weeks after initial inoculation [2]. As of April 2021, the US Food and Drug Administration (FDA) has issued six emergency use authorizations for treatments of confirmed mild to severe COVID-19 cases and three emergency use authorizations for vaccines [3].

Hepatic manifestations of COVID-19 infections have previously been documented. In one earlier study pertaining to extrapulmonary manifestations of COVID-19, it was noted that 14-53% of patients with COVID-19 had abnormally elevated levels of alanine aminotransferase (ALT) and aspartate aminotransferase (AST) [4]. Additionally, the degree of enzyme elevation is associated with more severe cases of COVID-19 [4-6]. The exact mechanism of this liver injury in the context of COVID-19 infection is not completely understood, but a few factors have been postulated to play a role. One mechanism is direct damage from viral replication within cells [5,7,8]. Based on prior studies of the coronavirus family, it has been demonstrated that the virus enters cells via the ACE2 receptor, which is expressed on multiple cells in the body including hepatocytes and bile duct epithelial cells [6,8]. Viral replication and release result in the rupture of cells, generating elevated liver enzymes in the serum [8]. Another process implicated in disease is immune-mediated damage secondary to the impressive cytokine storm triggered by the virus [5,7,8]. Immune-mediated damage has been accepted based on the fact that liver injury has been more common in patients with more deranged elevations in cytokines such as ferritin, LDH, C-reactive peptide, interleukin 6 and interleukin 2, and post-mortem liver biopsy showing inflammatory changes [6]. Additional mechanisms include drug-induced injury from treatments like hydroxychloroquine or lopinavir, and hypoxia from respiratory disease leading to hypoxic hepatitis [5,7,8]. 

Although hepatic injury can occur with COVID-19, few cases within the literature describe the initial presentation of the disease as acute hepatitis. Here we present a case of COVID-19 that presented with acute icteric hepatitis in the absence of underlying liver disease or severe respiratory symptoms.

## Case presentation

**Figure 1:**
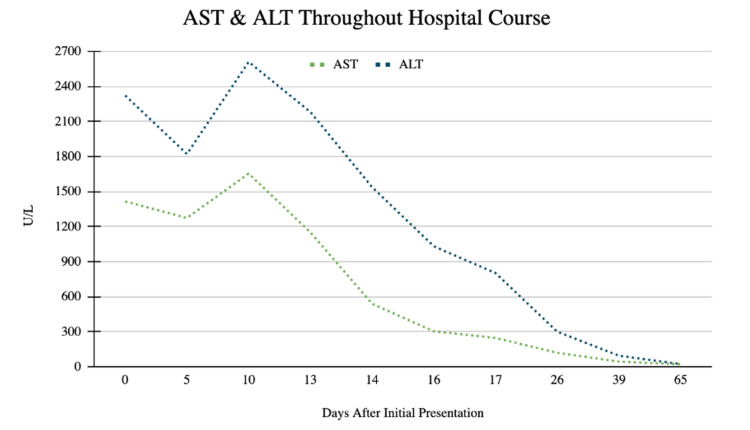
AST & ALT Throughout Hospital Course This image shows the trend of AST and ALT throughout hospital course in U/L. N-acetylcysteine was initiated on hospital day 12 while Vitamin K and Ursodiol were initiated on hospital day 14. Subsequent reductions in transaminases were observed as well as total bilirubin, direct bilirubin, and alkaline phosphatase (Figures 2-3).

**Figure 2:**
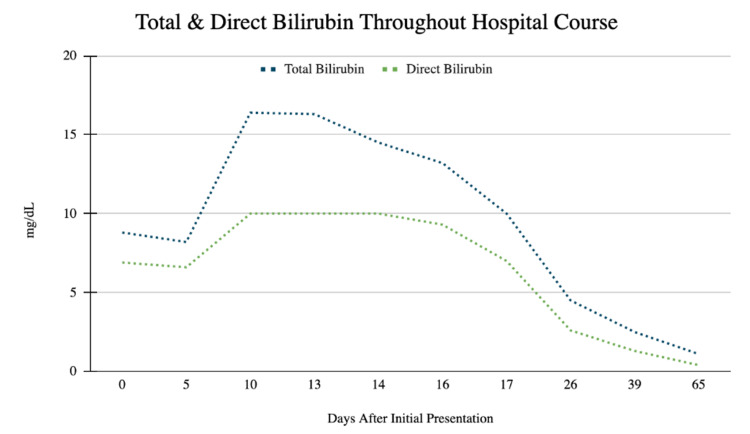
Total & Direct Bilirubin Throughout Hospital Course This image shows the trend of total and direct bilirubin throughout hospital course in mg/dL. Direct bilirubin levels of 10 mg/dL as seen on hospital days 10-14 were unable to be calculated and labeled as “>10 mg/dL” in the electronic medical record.

**Figure 3:**
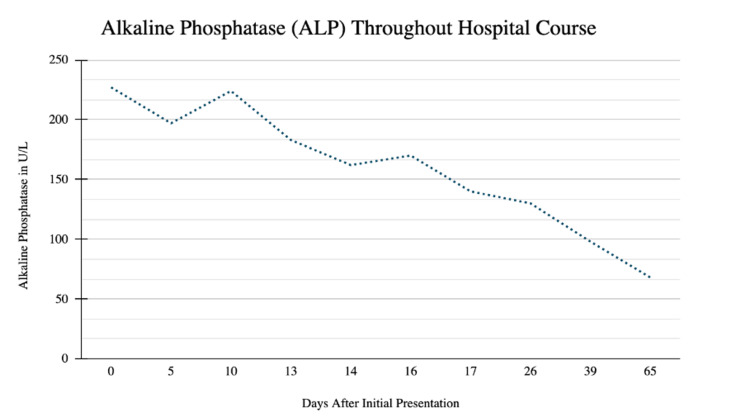
Alkaline Phosphatase (ALP) Throughout Hospital Course This image shows the trend of ALP levels throughout the hospital course in U/L.

A 29-year-old male with no past medical history presented to the emergency department with concerns of constipation for 10 days duration and subsequent development of scleral icterus five days later. The patient also reported early satiety during this time. He denied Tylenol or excess alcohol ingestion, history of hepatitis, IV drug use, gallbladder disease, or a history of transfusions. Notably, his wife tested positive for COVID-19 four days prior to presentation, and the patient had been quarantining for three days. He denied other sick contacts or recent travel. He reported a fever of up to 100.3 two days prior to presentation, mild sore throat with cough, chills, and fatigue. He denied shortness of air. He reported drinking alcohol socially once or twice a week and denied drinking since his symptoms began. He denied tobacco use and smoked marijuana occasionally, with the last use three months ago. His family history is negative for liver disease. His vital signs were significant for blood pressure 150/100 mmHg, temperature 100.3° F, heart rate 107 bpm, and oxygen saturation of 97% on room air. Physical exam showed appropriate mentation, scleral icterus, and epigastric tenderness to palpation; it was negative for hepatosplenomegaly, spider angiomata, or increased work of breathing, or any other stigmata seen in chronic liver disease. Complete metabolic profile and complete blood counts were significant for WBC 2.7 10^3/cmm, total bilirubin 8.8 mg/dL, direct bilirubin 6.9 mg/dL, indirect bilirubin of 1.9 mg/dL, AST 1415 U/L, ALT 2322 U/L, and alkaline phosphatase 227 U/L (Figures 1-3, Tables 1-3). Creatinine, albumin, platelet count, coagulation studies, and lipase were within normal limits. A COVID PCR was positive. He was admitted for further workup of liver enzyme abnormalities.

**Table 1:** AST & ALT Values Throughout Hospital Course For corresponding values, see Figure 1. Values are in U/L.

Days After Initial Presentation	AST	ALT
0	1415	2322
5	1273	1821
10	1653	2610
13	1150	2181
14	538	1537
16	302	1030
17	245	801
26	118	298
39	42	93
65	20	21

**Table 2:** Total and Direct Bilirubin Values Throughout Hospital Course For corresponding values, see Figure 2. Values are in U/L.

Days After Initial Presentation	Total Bilirubin	Direct Bilirubin
0	8.8	6.9
5	8.2	6.6
10	16.4	10
13	16.3	10
14	14.5	10
16	13.2	9.3
17	10	7
26	4.5	2.6
39	2.5	1.3
65	1.1	0.4

**Table 3:** Alkaline Phosphatase Values Throughout Hospital Course For corresponding values, see Figure 3. Values are in U/L.

Days After Initial Presentation	Alkaline Phosphatase
0	227
5	197
10	224
13	183
14	162
16	170
17	140
26	130
39	98
65	68

Throughout the patient’s hospital course, a comprehensive workup for acute hepatitis was completed. A right upper quadrant ultrasound was negative for acute cholecystitis, common bile duct dilation, and hepatic and portal vein thrombosis; the liver was noted to have normal echogenicity. MRCP showed diffuse hyperintensities along the periportal tract likely relating to acute hepatitis with mild fluid around the gallbladder fossa that was likely reactive to hepatocellular disease. Toxicology screening, including alcohol, acetaminophen, aspirin, and urine drug screen, was negative. Viral serologies were negative for hepatitis A, Β, C, acute CMV, acute EBV, HSV1, HSV2, HIV1 and HIV2. Hepatitis C RNA was undetectable. The autoimmune panel, which included anti-smooth-muscle antibody, antinuclear antibody, anti-double-stranded DNA, IgG, and anti-liver-kidney-microsomal antibody, was negative. Ceruloplasmin was normal. Iron studies showed elevated iron, percent saturation, and ferritin; however, genetic testing for hemochromatosis later resulted as negative for C282Y and H36D variants in the HFE gene. It was suspected that acute hepatitis was secondary to COVID-19 hepatitis. The patient remained on room air throughout his hospitalization. His sore throat resolved quickly after admission and he remained asymptomatic from a respiratory perspective thereafter. He did not require antivirals, steroids, monoclonal antibodies, or supplemental oxygen. Liver function test and coagulation studies were obtained daily and showed steady improvement (Figures 1-3, Tables 1-3). On the day of discharge, his labs were notable for a total bilirubin of 8.2 mg/dL, direct bilirubin 6.6 mg/dL, indirect bilirubin 1.6 mg/dL, AST 1273 U/L, ALT 1821 U/L, and alkaline phosphatase 197 U/L. He was instructed to repeat labs and follow up with hepatology the following week. 

Seven days following his discharge and 13 days after his initial presentation, repeat outpatient laboratory tests showed worsening liver function, with AST 1653 U/L and ALT 2610 U/L (Figures 1-3, Tables 1). The patient reported persistent jaundice, but denied symptoms of cough, shortness of air, anosmia, and ageusia. Vital signs were normal. The patient was mentating appropriately and had notable scleral icterus and jaundice. Repeat right upper quadrant US was stable from prior admission. The patient was readmitted for acute hepatitis.

**Figure 4:**
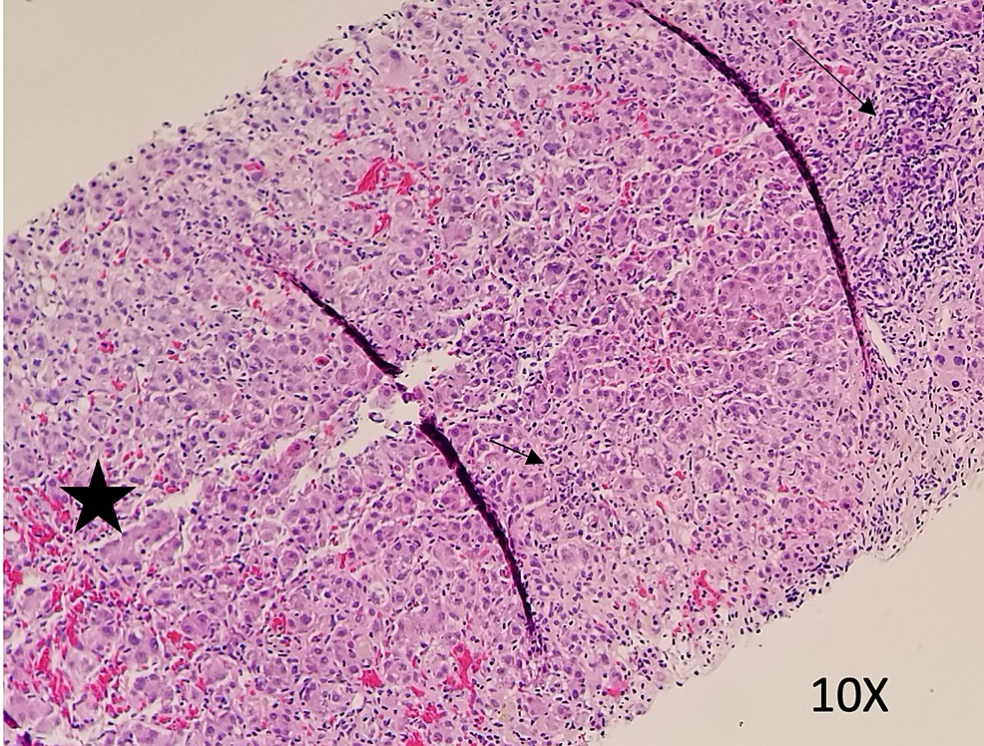
Liver Biopsy Liver biopsy showing periportal (long arrow) and lobular (short arrow) inflammation with centrilobular (star) congestion. No features of steatosis were identified. (10x)

**Figure 5:**
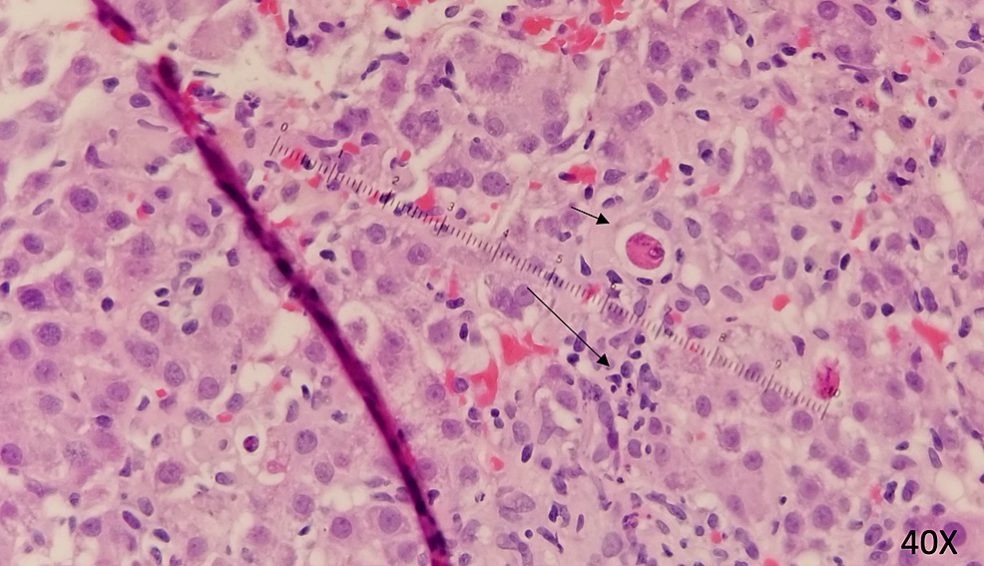
Liver Biopsy Liver biopsy showing hepatocyte resetting, spotty necrosis (long arrow) and apoptotic bodies (short arrow). No features of steatosis were identified. (40x)

The patient remained hospitalized for a total of nine days. On hospital day two, he was initiated on empiric treatment for liver injury with N-acetylcysteine 15mg/kg/hr IV infusion for a total of seven days of treatment. Starting on day three of hospitalization, he received three days of vitamin K 10mg IV daily for INR elevation and ursodiol 300mg three times per day PO for seven days. Liver biopsy was performed on day six of admission and was suggestive of acute hepatitis. Pathology demonstrated periportal and interstitial inflammation with predominantly lymphocytes, rare plasma cells, and neutrophils. Hepatocyte rosette formation, apoptotic bodies, and centrilobular congestion were also noted (Figures 4-5). Reticulin stain showed preserved liver architecture. Trichrome stain showed mild increased portal and pericellular fibrosis. PAS stain revealed normal hepatocyte cytoplasmic glycogen content that is completely digested with the PAS-diastase (PAS-D) stain. Prussian blue stain was positive for iron granules (1+/4+). Classic features of autoimmune hepatitis were not present and immunostains were negative for CMV, HSV1, and HSV2. No features of steatosis were identified on the biopsy. Liver function and coagulation tests were obtained daily and showed consistent downtrending starting on day five of admission (Figures 1-3, Tables 1-3). He was scheduled to follow up in three weeks and obtain repeat labs. 

Repeat outpatient liver function tests continued to improve. Three weeks after discharge and five weeks after his initial presentation, labs showed normalization of liver function tests, with AST 20 U/L and ALT 21 U/L (Figures 1-3, Tables 1-3). The patient is scheduled to undergo a repeat liver biopsy three months after resolution.

## Discussion

In summary, our patient presented with jaundice and mild symptoms of viral infection, including sore throat and fatigue, with hepatocellular and cholestatic patterns of injury discovered on laboratory testing. He did not develop overt signs of respiratory distress requiring supplemental oxygenation or intensive care unit level of management, nor did he develop liver failure. He received empiric treatment for hepatic dysfunction but did not receive antivirals, steroids, monoclonal antibodies or supplemental oxygen for COVID-19 infection. Viral, autoimmune, metabolic, toxic, and biliary causes of acute hepatitis were ruled out. In addition to the non-specific inflammatory findings on liver biopsy with negative stains, these findings further suggested acute hepatitis secondary to COVID-19 infection. However, as the patient did test positive for COVID-19, we did not collect a COVID-19 PCR on the liver biopsy itself. While this is a limitation, our extensive workup led to a diagnosis of exclusion for acute COVID-19 hepatitis. To our knowledge, there are no cases in the literature that document icterus and severe hepatic injury with cholestasis as the primary presentation of COVID-19. This case also defies the accepted association of disease severity with the level of aminotransferase elevation, as although the patient had severe liver injury, he did not require oxygen or intensive care level management. 

Only a few cases of asymptomatic COVID-19 presenting with acute hepatitis were found in the literature [9-11]; there are even fewer cases of severe liver disease in patients without a history of pre-existing liver disease. Wander et al. presented an acute case of non-icteric hepatitis in a woman with human immunodeficiency virus; however, although she initially lacked respiratory symptoms, she developed symptoms hours after the diagnosis of COVID-19 and required supplemental oxygen [10]. Additionally, the patient had multiple contributors to elevated transaminases including her medications and medical comorbidities. Bongiovanni and Zago also presented an acute case of COVID hepatitis in a female without respiratory symptoms or medical comorbidities, which is more similar to our patient [9]; notably, the patient did not have jaundice and had normal serum bilirubin level of 1.02 mg/dL. Our case adds to the literature another example of this rare presentation of COVID-19- acute hepatitis presenting without respiratory symptoms. Additionally, it is the first to our knowledge to show acute hepatitis with jaundice in the absence of respiratory symptoms. 

Hepatocellular patterns of injury have been documented in patients with hepatitis from COVID-19; cholestatic patterns are less common [5,7-9]. Slight increases in bilirubin are seen in only 10% of cases, and elevations in other markers of cholestasis like alkaline phosphatase or gamma-glutamyl-transferase (GGT) remain infrequent [8]. Our patient presented with scleral icterus and jaundice with total bilirubin elevated to 8.4 mg/dL on first admission and 16.4 mg/dL on subsequent admission. Additionally, our patient also had elevations in alkaline phosphatase and GGT close to 2x the upper normal limit (UNL). It is unclear if these elevations in cholestatic markers are reactive to ongoing hepatitis or caused by direct viral damage of biliary epithelial cells. Interestingly, the ACE2 receptor by which the virus enters cells for replication is more robustly expressed on biliary epithelial cells than hepatocytes [7]; therefore, it would seem that cholestatic patterns would predominate over hepatocellular patterns of injury. However, this is not what has been described in the literature, and may suggest that other receptors may be implicated in disease. Further studies are required to better characterize the mechanism of cholestasis in acute COVID hepatitis. 

The severity of COVID-19 has been associated with elevated liver enzymes. In a retrospective study of PCR positive COVID patients with liver function testing across three hospitals in the New York-Presbyterian system, researchers used alanine aminotransferase (ALT) derangements to classify the extent of liver disease in those with COVID-19 to better characterize clinical outcomes [5]. The patients’ liver disease was classified as mild if ALT was elevated but <2x UNL, moderate if 2x<ALT<5x UNL, and severe if ALT >5x UNL [5]. In comparison to those with mild and moderate liver injury, patients with severe liver injury were more likely to require intensive care unit (ICU) level care, intubation, and renal replacement therapy; they also had a greater risk of in-hospital mortality [5]. Using this paradigm, our patient meets the criteria for severe liver disease. However, he remained asymptomatic from a respiratory perspective and never developed organ failure requiring advanced therapies. This benign clinical course in the setting of severe lab derangements is perplexing. 

Other studies have associated elevations in AST and AST/ALT ratios >2 with increased rates of ICU admissions [12]. It is important to understand however that AST is emitted from multiple tissues and therefore, elevations in AST are not specific for liver injury; instead, severe elevations in AST may represent more multi-organ involvement [12]. With this understanding, we believe that elevated AST/ALT ratios may represent more systemic inflammation, while lower AST/ALT ratios may be more specific for hepatic injury. Of note, although our patient had a significant elevation in AST, his AST/ALT ratio was low at 0.63. This may suggest that he had a less severe ongoing systemic inflammatory response and may explain his benign course of COVID-19. The exact mechanism and predilection of hepatic manifestations in COVID-19 infections is still unknown and requires further exploration.

## Conclusions

In conclusion, we describe a patient with initial presentation of COVID-19 as acute icteric hepatitis without respiratory symptoms, which has not been described within the literature to our knowledge until now. The jaundice and cholestatic pattern of hepatic injury in this case suggests that biliary damage may be more implicated than previous studies have suggested. Additionally, this case contraindicates the association of severity of coronavirus infection with a degree of aminotransferase elevation; although the patient had severe liver injury, he remained asymptomatic from a respiratory standpoint and did not require antivirals, steroids, or supplemental oxygen. Further studies are required to better characterize the mechanism of liver injury and pattern of injury so that physicians are better equipped to recognize COVID-19 in patients who do not present with typical symptoms.
